# Evaluation of lentinan effects on cytochrome P450 activity in rats by a cocktail method

**DOI:** 10.22038/ijbms.2019.31611.7611

**Published:** 2019-03

**Authors:** Yiping Lin, Yanli Wei, Xiaoxia Hu, Meiling Wu, Jingchan Yao, Xiaoqian Ying, Xiaoyan Fu, Mingxing Ding, Liman Qiao

**Affiliations:** 1 Jinhua Polytechnic, Jinhua 321007, Zhejiang, China; 2 Jinhua Central Hospital, Jinhua 321000, Zhejiang, China; 3 The Second Affiliated Hospital and Yuying Children’s Hospital of Wenzhou Medical University, Wenzhou 325000, Zhejiang, China

**Keywords:** Cocktail, CYP, Herb-drug interaction, Lentinan, Probe drug

## Abstract

**Objective(s):**

In this study, a cocktail of probe drugs was used to assess whether lentinan could influence the activities of rat enzymes CYP3A4, CYP2D6, CYP1A2, CYP2C19, and CYP2C9 in vivo.

**Materials and Methods:**

Fourteen days after intraperitoneal injection of lentinan, rats were given an oral dose of a cocktail solution containing phenacetin, tolbutamide, omeprazole, metoprolol, and midazolam. Then, we obtained blood in specific durations for the determination of plasma concentration of the probe drugs using UPLC-MS/MS. We also evaluated the pharmacokinetic parameters using the DAS 2.0 software.

**Results:**

We found that various concentrations of lentinan increased the activity of rat CYP1A2, CYP3A4, CYP2D6, and CYP2C19 but not CYP2C9.

**Conclusion:**

These findings suggest that clinical application of lentinan combination with CYP3A4, CYP1A2, CYP2C19, or CYP2D6 should be given careful consideration as this may lead to herb-drug interactions and hence treatment failure.

## Introduction

Lentinan is an immunostimulant polysaccharide extracted from the *Lentinula edodes *mushroom, which is made up of β-(1→6) branched β-(1→3)-glucan. Lentinan inhibits primary, allogeneic transplanted, and the xenografted tumors (1). Furthermore, it can enhance the effectiveness of chemotherapy drugs and reduce the toxicity (2). Moreover, lentinan has minimal side effects (3). Since 1995, lentinan was applied in-clinic for the treatment of malignant tumors as an immunotherapeutic agent in China (4). Now, there are more than six kinds of lentinan injections used in-clinic (4).

Cytochrome P450 (CYP) enzymes comprise approximately 70–80% of enzymes involved in drug metabolism in humans (5, 6). Among them, CYP3A4, CYP2D6, CYP2C19, CYP2C9, and CYP1A2 regulate the metabolism of many drugs (7). These isozymes show different interindividual variations, and their activities can be largely affected by herb medicines (8). Importantly, it has been observed that simultaneous use of prescribed drugs with those of herbal origin may result in drug interactions due to activation or inhibition of CYP. 

Therefore, investigating the effect of herbal drugs on CYP enzyme activities is essential for clinical prediction of herb-drug interactions, investigation of potential toxic effects, and explaining inter-subject variability (9). Recently, a “cocktail” approach is widely used, because it could obtain information about multiple CYP activities of many pathways in a single experiment (10). Thus, various cocktail methods have been developed and used to study possible drug interactions and the effect of herbal drugs on CYP activities (11-13).

Lentinan is an herbal drug that is used as an antitumor in-clinic. However, its effect on rat CYP enzyme activities has not been evaluated. Here, we assessed whether lentinan could influence the activities of rat CYP3A4, CYP2D6, CYP2C19, CYP2C9, and CYP1A2 enzymes. We anticipate that the findings of our study would shed more light on the safety of lentinan based herbal drugs in term of drug interactions.

***Experiments***

***Chemicals and reagents***

Lentinan (> 98%) used in this study was obtained from Shanxi Taisheng Pharmaceutical Co., Ltd. (Shanxi, China). Phenacetin, tolbutamide, omeprazole, metoprolol, midazolam (all > 98%), and the internal standard (IS) carbamazepine were obtained from Sigma-Aldrich Company (St. Louis, MO, USA). LC grade methanol and acetonitrile were obtained from the Merck Company (Darmstadt, Germany). All reagents were of analytical grade and were utilized with no additional purification. The Milli Q water purification system (Millipore, Bedford, USA) was used to prepare the water used for the LC-MS/MS analysis. 

***Chromatographic analysis***

 Liquid chromatography was performed on an Agilent UHPLC unit (Agilent Corporation, MA, USA) with a ZORBAX Eclipse Plus C18 column (1.8 μm, 2.1× 50 mm). The mobile phase comprised solution A (0.1% formic acid in water) and solution B (acetonitrile), and a gradient program was employed as follows: 30% B (0-0.3 min), 30-50% B (0.3-1.3 min), 50-95% B (1.3-1.8 min), 95-95% B (1.8-2.8 min), 95-30% B (2.8-3.0 min), 30-30% B (3.0-4.0 min). The injection volume was 5 μl and the flow rate was 0.40 ml/min. The temperature of the column was kept at 30 ^°^C. 

Mass spectrometric analysis was performed using Agilent 6420 triple-quadrupole mass spectrometer equipped with an electrospray ionization source (Agilent Corporation, MA, USA). Multiple reaction monitoring (MRM) mode was used for quantitation. The precursor-product ion pairs used for the MRM detection were m/z 180.1 → 109.9 for phenacetin, m/z 271.11 → 91.0 for tolbutamide, m/z 346.1 → 135.9 for omeprazole, m/z 268.2 → 115.9 for metoprolol, m/z 326.1 → 290.8 for midazolam, and m/z 237.1 → 194.2 for carbamazepine (IS). The Agilent 6420 Quantitative Analysis version B.07.00 analyst data processing software (Agilent Corporation, MA, USA) was used for instrument operation and data acquisition.

***Animals and administration dosage***

 All procedures and experiments in this study were performed using 180–220 g weighing male Sprague-Dawley rats obtained from Laboratory Animal Center of Wenzhou Medical University (Zhejiang, China), and authorization was provided by the Animal Care and Use Committee of Wenzhou Medical University. Animals were handled in accordance with the guidelines for the Care and Use of Laboratory Animals. Rats were housed at 25 ± 1 ^°^C conditions with 12 hr light-dark cycle and provided with tap water *ad libitum* as well as standard rodent chow food. Rats were stabilized for one week before experiments.

Thirty-six rats were assigned into 6 groups (n=6 per group): blank control group (BCG), low dose test group (LTG), medium dose test group (MTG), high dose test group (HTG), induction group (IDG), and inhibition group (IHG). BCG was given saline by intraperitoneal injection for 14 consecutive days, while LTG, MTG, and HTG were administered lentinan at doses of 0.05, 0.2, and 0.5 mg/kg, in that order. IDG and IHG were administered phenobarbital and cimetidine at doses of 50 mg/kg, respectively by intraperitoneal injection for 14 days. On the 15th day, six groups were all orally given cocktail solutions simultaneously, which contained omeprazole (20 mg/kg), tolbutamide (1.0 mg/kg), phenacetin (20 mg/kg), metroprolol (20 mg/kg), and midazolam (20 mg/kg). At 0.167, 0.5, 1, 1.5, 2, 3, 4, 6, 9, 12, and 24 hr after oral administration of probe drugs, blood was obtained (0.3 ml) via the tail vein and put into heparinized polythene tubes. This was followed with centrifugation at 4000 g for 8 min to obtain 100 μl serum, which was stored at -20 ^°^C until analysis. 

***Sample preparation***

Two-hundred µl of the IS working solution (20 ng/ml in acetonitrile) was added to 100 µl of serum in a 1.5 ml centrifuge tube. The samples were mixed by vortexing for 1 min and then centrifuged at 13,000 g for 10 min. Analysis was performed by injecting the supernatant (2 µl) into the UPLC-MS/MS system.

***Statistical analysis***

 The DAS (Drug and statistics) software (version 2.0, Shanghai University of Traditional Chinese Medicine, China) was used to determine the plasma concentration of the drugs at each time-point. SPSS 19.0 (Chicago, IL) was used to determine statistical significance using t-test. *P*<0.05 was considered to be statistically significant.

## Results

In this study, the activities of CYP enzymes were evaluated following treatment of rats with a drug cocktail comprising: midazolam for CYP3A4, metoprolol for CYP2D6, omeprazole for CYP2C19, tolbutamide for CYP2C9, and phenacetin for CYP1A2 using a validated UPLC-MS/MS method. 

***Effect of lentinan on rat CYP1A2 activity***

**Figure 1:**
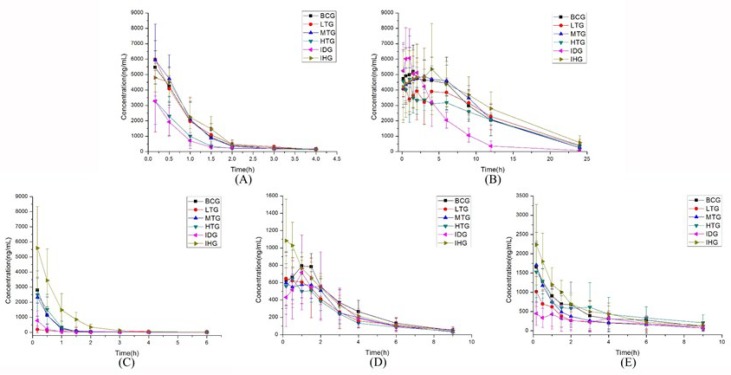
Time-concentration curves for phenacetin (A), tolbutamide (B), omeprazole(C), metoprolol (D), and midazolam (E), after various doses of lentinan in rats (Mean±SD, n=6) blank control group (BCG), low dose test group (LTG), medium dose test group (MTG), high dose test group (HTG), induction group (IDG), and inhibition group (IHG)

CYP1A2 activity was determined by comparing pharmacokinetic behaviors of phenacetin between different treated groups. Table 1 shows the key pharmacokinetic features of phenacetin while Figure 1A shows the mean plasma concentration-time curves of phenacetin in various groups. Table 1 shows that after high dose lentinan treatment in HTG, AUC_(0→t)_, AUC_(0-∞)_, t_1/2_, and C_max_ of phenacetin were decreased significantly compared to those of BCG. CL (plasma clearance) of phenacetin was remarkably elevated (*P*<0.05). These findings showed that the metabolic rate of phenacetin was enhanced in the treatment groups, and that lentinan may increase the activity of rat hepatic CYP1A2 *in vivo*; these changes in HTG were not significantly different from the IDG group. 

***Effect of lentinan on rat CYP2C9 activity***

The activity of CYP2C9 was evaluated based on the pharmacokinetic behaviors of tolbutamide following lentinan treatment. Table 2 shows the effect of various lentinan treatments on pharmacokinetic parameters of tolbutamide in rats while Figure 1B shows the mean plasma concentration-time curves of tolbutamide in the groups. No significant difference was found in terms of the pharmacokinetic parameters of tolbutamide (AUC_(0→t)_, AUC_(0-∞)_, t_1/2_, T_max_, C_max_, and CL) between lentinan and BCG groups. These observations implied that lentinan did not affect the activity of rat hepatic CYP2C9 *in vivo*.

***Effect of lentinan on rat CYP2C19 activity***

To evaluate the activity of CYP2C19, we compared the pharmacokinetic profiles of omeprazole between the groups. The results of the analysis are shown in Figure 1C and Table 3 in terms of mean plasma concentration-time curves and pharmacokinetic parameters of omeprazole. The AUC_(0→t)_, AUC_(0-∞)_, and C_max_ of omeprazole in LTG was lower than that of the BCG group, CL of omeprazole was increased significantly (*P*<0.05). No observable differences were noted on pharmacokinetic features between LTG and IDG. The findings showed that lentinan could not induce the activity of rat hepatic CYP2C19 in LTG. Interestingly, as the dose of lentinan increased, AUC_(0→t)_, AUC_(0-∞)_, t_1/2_, T_max_, C_max_ and CL of omeprazole in MTG and HTG showed no obvious changes from the BCG group. Therefore, the CYP2C19 activity decreased as the lentinan dose increased.

***Effect of lentinan on rat CYP2D6 activity***

The pharmacokinetic profile of metoprolol in rats treated with various doses of lentinan is shown in Table 4. Figure 1D shows the mean plasma concentration-time curves of metoprolol in the groups. AUC_(0→t)_ and AUC_(0-∞)_) of metoprolol decreased at the end of treatment with increasing doses of lentinan (*P*<0.05), and even smaller than those of IDG. However, other pharmacokinetic parameters of metoprolol indicated no obvious changes. In addition, it showed no significant differences between various lentinan groups and BCG. Hence, the pharmacokinetic properties of metoprolol suggested that lentinan could induce the activity of rat hepatic CYP2D6.

**Table 1:** Main pharmacokinetic parameters of phenacetin after various administrations of lentinan in rat plasma (Mean ± SD, n=6)

Parameter	BCG	LTG	MTG	HTG	IDG	IHG
AUC_(0→t)_(µg·h/L)	5,228.09±1,600.65	5,450.91±1,768.68	5,464.83±1,847.83	2,868.65±1,016.64*	2,577.29±1,319.78*	5,564.61±1,651.49
AUC_(0-∞)_ (µg·h/L)	5,915.48±1,520.96	5,812.90±1,371.13	5,575.40±1,809.10	3,558.85±1,089.91*	3,674.03±1,762.65*	5,653.25±1,629.60
t_1/2 _(h)	1.82±2.06	1.64±1.73	1.85±1.38	1.06±1.21*	0.74±0.93*	1.67±1.21
T_max_ (h)	0.22±0.14	0.17±0.00	0.17±0.00	0.22±0.14	0.23±0.15	0.39±0.17
CL/F (L/h/kg)	3.57±0.88	3.60±0.86	4.06±1.84	6.11±1.95*	7.40±5.68*	3.77±1.01
C_max_ (ng/mL)	5,724.59±756.51	5,990.43±1,201.65	5,935.16±2,326.59	3,471.83±1,346.76*	3,308.96±1,933.08*	5,001.48±815.57

* Significantly different from control, *P*<0.05.

**Table 2:** Main pharmacokinetic parameters of tolbutamide after various administrations of lentinan in rat plasma (Mean ± SD, n=6)

Parameter	BCG	LTG	MTG	HTG	IDG	IHG
AUC_(0→t)_(µg·h/L)	60,211.83±22,700.71	56,736.97±9,964.9	62,125.12±10,680.61	50,729.01±6,407.87	33,060.80±9,530.00	70,448.06±28,605.50
AUC_(0-∞)_ (µg·h/L)	61,934.99±23,746.90	62,219.69±10,111.76	64,085.42±10,294.90	61,356.00±11,822.75	33,610.18±9,950.91	78,357.87±41,163.60
t_1/2 _(h)	4.21±0.69	5.74±4.60	4.31±1.12	8.66±3.99	2.73±1.34	5.71±2.40
T_max_ (h)	2.00±1.67	2.52±1.92	1.85±1.45	0.63±0.49	1.42±1.32	3.81±1.65
CL/F (L/h/kg)	0.02±0.01	0.02±0.00	0.02±0.00	0.02±0.00	0.03±0.01	0.02±0.01
C_max_ (ng/mL)	5,294.74±1,776.37	5,085.80±1,311.57	5,278.29±968.57	6,254.46±3,082.64	6,224.87±1,851.25	5,584.03±2,831.18

**Table 3:** Main pharmacokinetic parameters of omeprazole after various administrations of lentinan in rat plasma (Mean ± SD, n=6)

Parameter	BCG	LTG	MTG	HTG	IDG	IHG
AUC_(0→t)_(µg·h/L)	1,406.44±516.32	337.16±220.80*	1,319.30±810.33	1,546.14±659.98	351.43±231.19*	4,471.65±2,419.93
AUC_(0-∞)_ (µg·h/L)	1,419.65±517.20	345.60±278.82*	1,334.18±805.76	1,564.24±658.86	357.21±230.39*	4,485.23±2,413.24
t_1/2 _(h)	1.58±1.16	1.51±1.34	1.37±1.10	1.37±0.67	1.35±1.05	0.79±0.36
T_max_ (h)	0.17±0.00	0.15±0.12	0.17±0.00	0.31±0.30	0.22±0.13	0.17±0.00
CL/F (L/h/kg)	16.88±9.70	53.14±26.24*	18.64±7.21	14.71±5.51	51.58±31.51*	6.11±3.73
C_max_ (ng/mL)	2,797.86±861.67	301.00±221.09*	2,325.45±1,257.45	2,705.32±1,297.50	777.83±651.99*	5,593.95±2,746.37

* Significantly different from control, *P*<0.05.

**Table 4:** Main pharmacokinetic parameters of metoprolol after various administrations of lentinan in rat plasma (Mean ± SD, n=6)

Parameter	BCG	LTG	MTG	HTG	IDG	IHG
AUC_(0→t)_(µg·h/L)	2,800.68±523.17	2,156.11±894.15*	2,171.48±510.65*	1,886.15±448.48*	2,307.79±1,153.49*	2,803.60±443.43
AUC_(0-∞)_ (µg·h/L)	2,934.20±541.74	2,313.78±1,007.34*	2,256.67±532.51*	2,110.02±462.41*	2,446.33±1,132.02*	2,967.64±411.93
t_1/2 _(h)	1.96±0.64	2.26±0.54	1.83±0.25	2.10±2.08	1.88±0.84	2.29±0.65
T_max_ (h)	0.92±0.51	0.71±0.43	0.81±0.63	0.52±0.24	1.07±0.19	0.29±0.17
CL/F (L/h/kg)	7.02±1.31	10.84±6.11	9.40±2.62	9.99±2.81	9.19±2.65	6.87±1.06
C_max_ (ng/mL)	864.75±142.32	795.86±349.73	762.12±227.69	680.15±321.96	720.91±428.50	1,221.57±385.50

* Significantly different from control, *P*<0.05.

**Table 5:** Main pharmacokinetic parameters of midazolam after various administrations of lentinan in rat plasma (Mean ± SD, n=6)

Parameter	BCG	LTG	MTG	HTG	IDG	IHG
AUC_(0→t)_(µg·h/L)	3,967.78±2,375.33	2,401.19±1,617.94*	2,873.79±1,417.22*	4,421.88±2,840.97	1,729.69±1,739.39*	4,802.16±1,678.29
AUC_(0-∞)_ (µg·h/L)	4,533.65±3,013.80	3,016.53±2,570.46*	3,238.62±1,755.94*	4,606.62±4,590.78	2,286.88±2,658.33*	5,214.14±1,995.20
t_1/2 _(h)	2.56±1.30	2.90±2.42	2.45±1.59	2.42±2.71	2.43±2.26	2.01±1.09
T_max_ (h)	0.74±0.87	0.31±0.30	0.32±0.13	0.56±0.99	0.17±0.49	0.21±0.12
CL/F (L/h/kg)	6.46±4.00	15.14±18.87*	7.89±3.90	6.84±6.41	53.90±56.20*	4.95±3.71
C_max_ (ng/mL)	1,771.77±521.69	1,076.98±447.53*	1,745.45±791.59	1,759.36±683.93	512.70±359.03*	2,359.63±1,014.58

* Significantly different from control, *P*<0.05.

***Effect of lentinan on rat CYP3A4 activity***

The pharmacokinetic characteristics of midazolam were compared between the control and lentinan treatment groups to determine the CYP3A4 activity. Table 5 illustrates the influence of various lentinan treatments on the pharmacokinetic profile of midazolam. Figure 1E is a presentation of the mean midazolam plasma concentration-time curves of the groups. Following pretreatment with the low dose of lentinan the C_max_, AUC_(0→t)_, and AUC_(0-∞)_ of midazolam in LTG decreased compared to the BCG group. CL of midazolam was enhanced remarkably (*P*<0.05). However, as the dose increased, these changes become less pronounced. Therefore, CYP3A4 activity revealed that, at low dose, lentinan increased the activity of enzyme while at high dose, it decreased the enzyme activity.

## Discussion

The CYP enzymes are involved in drug metabolism in many vertebrate species. Some of the key functions of these classes of enzymes include the breakdown of several xenobiotics and endogenous compounds as well as modulation of peroxidative, reductive, and oxidative processes. To aid the study of CYP isozyme activities, several probe drugs have been designed using the ‘‘cocktail’’ approach. This maneuver is particularly important because it prevents analytical or metabolic interactions between the drugs (14). Previously, we designed a probe-drug cocktail comprising midazolam, tolbutamide, phenacetin, omeprazole, and metoprolol to facilitate the UPLC-MS/MS-based analysis of five probe drugs in one experiment. This technique can be applied to study the activity of CYP3A4, CYP1A2, CYP2C9, CYP2C19, and CYP2D6. In this study, the effect of lentinan on the activities of these CYP isozymes was investigated in rats.

Out of the total CYP content in human liver, CYP1A2 constitutes 13% (15) and is an important player in the metabolic processing of drugs and many other endogenous compounds. It is also known to exert procarcinogenic effects by activating aflatoxin B1, a major hepatocarcinogen (16). Based on our results, CYP1A2 activity was enhanced in HTG. It is possible that when the dose of lentinan increased, the metabolic capacity was built up and increased so that the effect was induced CYP1A2. However, the precise mechanisms that account for these observations remain elusive.

As we know, CYP2C9 constitutes ~20% of hepatic total CYP content. About 15% of clinical drugs (>100 drugs) are metabolized by this enzyme, including those that have a small therapeutic range (17). Therefore, an increase or decrease of the activity of CYP2C9 will greatly affect the therapeutic effect of many drugs. Here, the results showed that lentinan administration did not alter the pharmacokinetic characteristics of tolbutamide. Hence, this implies that lentinan did not activate or inhibit the CYP2C9 activity in rats.

 CYP2C19 facilitates the metabolism of mephenytoin, diazepam, and proton pump inhibitors (18). Furthermore, CYP3A4 is considered to be a rate-limiting step in the catabolism and clearance of many clinical drugs, e.g., pediatric drugs (19). In our study, investigation of CYP2C19 and CYP3A4 activities revealed that, when the dose of lentinan was increased, its effect on the enzyme activity changed to inhibitory. Notably, low dose of lentinan could induce CYP2C19 and CYP3A4 activities in rats. These findings demonstrate that lentinan may have herb-drug interactions when combined with other drugs, especially those metabolized by CYP2C19 and CYP3A4. Hence, care should be taken when using this drug to avoid treatment failure as a result of low drug plasma concentration.

CYP2D6 has been extensively researched and it constitutes 2–4% of total hepatic CYP content. It is involved in the metabolism of many drugs, especially those targeting cardiovascular diseases and the CNS. Some of these kinds of drugs have a narrow therapeutic index (20, 21). Our results showed that the positive effect of lentinan on CYP2D6 increased with the dose. The differences among different doses of lentinan suggested that the subtype of enzymes affected correlated with the dose of administration. Taken together, our findings demonstrate that lentinan may induce drug interactions when co-administered with drugs metabolized by CYP2D6. In the case of drugs with narrow therapeutic windows, care should be taken when combining them with lentinan.

## Conclusion

Our study has revealed that lentinan does not induce clinically relevant herb-drug interactions based on CYP2C9 activity analysis. In line with our findings, we found that comedication of lentinan with drugs metabolized by CYP1A2, CYP2C19, CYP2D6, and CYP3A4 may compromise the metabolism of these drugs and decrease their plasma concentrations. Additional clinical investigations are warranted to explore the safety of lentinan for clinic use. 
